# Surgical Nursing

**Published:** 1899-09

**Authors:** 


SURGICAL NURSING. By BERTHA M. VOSWINKEL, Graduate of Episcopal Hospital, Philadelphia; late nurse-in-charge of Children's Hospital, Columbus, O. Second edition, revised and enlarged, with 112 illustrations. Philadelphia; P. Blakiston's Son & Company: 1899.

This work on surgical nursing is in its second edition, a fact which is of itself a recommendation for the book. It is plainly written, concise and well illustrated. Barring a few rather careless typographical errors, there is nothing to criticise, and it strikes the reviewer as being a work which the nurse who felt her training deficient in the care of surgical cases would find very helpful.

